# Cases of Gonorrhœa, Successfully Treated by Cubebs

**Published:** 1821-06

**Authors:** Miles Marley

**Affiliations:** Member of the Royal College of Surgeons.


### *Cases of Gonorrhœa, successfully treated by Cubebs.* By Miles Marley, Member of the Royal College of Surgeons.

As we are daily hearing contradictory reports respecting the efficacy of cubebs in cases of gonorrhœa, and as I have given that medicine fair and repeated trials, I think I do no more than my duty in laying the results of my experience before the profession. I must here intimate that it has been my aim to compress the substance of this communication into as small a compass as possible, and to state nothing respecting the remedy that might undeservingly enhance its reputation. I have written it with a mixed feeling of confidence and distrust, hoping that it may apologize for itself by its good intention,—namely, that of strongly recommending a medicine capable of quickly curing one of the most frequent disorders that comes under the practitioner's care; and at the same time, fully aware of the opposition the remedy has already met with, I do not mean to assert that the medicine under consideration is an infallible remedy in all cases, but candidly acknowledge that it has seldom or never answered my expectations in gleets, or in cases of above a month's standing. In cases of a recent nature, I think I may call it a specific. Much depends upon the medicine being genuine, which I am inclined to think is not always the case; and to that circumstance I, in a great degree, attribute the failures which have occurred in the use of it. The dose is likewise a matter of importance: I never give less than from one to three drachms three times a-day, having found that smaller doses are of no service. During the use of the medicine, it is necessary to restrict the patient to the antiphlogistic regimen. In some instances I have been led to think that small doses of calomel expedite the good effects of cubebs; and, with this view, in cases not of a very recent nature, I generally give a couple of grains at bed-time, about every other night. The head and stomach are always more or less affected during the exhibition of cubebs, and there is generally a tendency to constipation, which must be obviated by some of the neutral salts. I mostly desire it to be taken in milk; but, should the stomach prove too irritable to retain it in that form, coffee will be found an excellent vehicle. To guard against a relapse, I always continue the use of the medicine three or four days after the disease has disappeared.

CASE I.

*Jan.* 28*th*, 1821.—Robert Lincoln, aged 24, had connexion about nine days ago; complains of considerable pain in making water, and has done so for the last four days; he says, he has had scarcely any sleep of late, owing to chordee. He perceived the discharge on the fourth day: it was white, and of a thin consistence, and has remained so to this time. Let him take of powdered cubebs, ʒij. three times a-day.

30*th*.—No pain on expelling his urine, nor has he been troubled with chordee. The discharge not altered.

31*st*.—The discharge much thicker. Continue as before.

*Feb.* 2*d*.—The discharge almost gone. Continue.

4*th*.—Says the discharge stopped yesterday, (the 3d,) and has not since returned. Continue for two days longer.

CASE II.

*Dec.* 14*th*, 1820.—A young gentleman, aged 19, had connexion eight days ago; yesterday perceived a discharge of a purulent yellowish matter, slightly tinged with blood, accompanied with a tickling sensation in the course of the urethra. To-day he complains of much pain on making water. Let him take of powdered cubebs, ʒij. three times a-day.

15*th*.—Little or no alteration. Continue.

16*th*.—Complains of a slight heat, not amounting to pain, in the urinary passage; the discharge is thicker. Continue.

18*th*.—No unpleasant sensation in the urethra, and the discharge is lessened considerably. Continue.

20*th*.—No symptom of the disease now remains. Continue for two days longer.

CASE III.

*Oct.* 19*th*, 1820.—A man, aged 24, consulted me for a discharge of a thin yellowish matter. He had connexion a fortnight ago; perceived the discharge about five days after; thinks it increases in quantity every day; complains of much pain on making water, and has had chordee for the last three nights. Says he has had the disease five or six times, and has never been cured in less than two months or six weeks. Let him take of powdered cubebs, ʒiij. three times a-day in milk; and of calomel, two grains, every other night at bed-time.

20*th.*—Took but one of the powders yesterday, his stomach being irritable. Continue; let the medicine be taken in coffee.

22*d.*—The powders have been retained on the stomach; no ardor urinæ; the discharge is thicker, and less abundant.

24*th.*—I was not a little astonished to find my patient free from all symptoms of the disease. Continue for three or four days longer.

CASE IV.

*Oct.* 26*th*, 1820.—A gentleman's groom, 22 years of age, has had a discharge of purulent matter from the urethra for the last five days; it does not seem to increase in quantity; perceived nothing unpleasant till yesterday, when he felt a scalding pain on expelling his urine, accompanied with redness and swelling at the end of the urethra. Let him take of powdered cubebs, ʒij. three times a-day, and apply cold lotion to the penis.

27*th.*—The discharge unaltered; the swelling, redness, and ardor urinæ, much diminished. The left testicle is nearly twice its natural size; which I attribute not to the medicine, but to the patient having taken a long rough-ride yesterday afternoon. Continue the powders as before. Support the testicle by means of a suspensory bandage, and apply cold lotion.

29*th.*—The swelling of the testicle has completely subsided; complains of nothing now but the discharge, which is gradually diminishing.

*Nov.* 3*d.*—Is quite well. Continue two days longer.

CASE V.

*Jan.* 25*th*, 1821.—Mr. L— was infected six days ago; did not perceive any thing unpleasant till yesterday morning, when he discovered a discharge of a straw-coloured matter, accompanied with a scalding on making water. Has never had this form of the disease before, but had chancres some time since, for which he took mercury, and was cured in the course of six weeks. Let him take of powdered cubebs, ʒiij. three times a-day, in milk.

26*th.*—Complains much of nausea, and begs of me to change the medicine. The discharge lessened, and of a thicker consistence. Continue to take the powders in coffee.

29*th.*—Is perfectly free from the disorder. Continue four days longer.

CASE VI.

*Feb.* 27*th*, 1821.—P. H. esq. aged 25, had connexion nine days ago; three days after, he found an abundant discharge of yellowish matter, which has continued ever since; complains of pain and difficulty in making water; is troubled much with painful erections, and a blush of inflammation surrounds the orifice of the urethra. Take of cubebs in powder, ℨiij. three times a-day. Apply cold lotion to the penis.

*29th.*—No pain on making water; has been but little troubled with chordee; the discharge is much the same in quantity, but begins to assume a thicker appearance. Continue.

*31st.*—The discharge is almost gone. Continue.

*March 2d.*—Cured. Continue two days longer.

CASE VII.

*Jan. 27th,* 1821.—A man, aged 35, has had a discharge of purulent matter from the urethra for the last four or five days; he occasionally complains of slight pain on making water. I desired him to take ℨiss. of powdered cubebs three times a-day.

*30th.*—The discharge is scarcely perceptible; no pain on making water.

*Feb. 1st.*—The complaint is completely removed. Continue two days longer.

CASE VIII.

*Nov. 24th,* 1820.—A gentleman, 40 years of age, had connexion five days ago; complains of scalding on making water, with much difficulty in retaining it; has a profuse discharge of purulent matter, with slight excoriation of the glans. Let him take of powdered cubebs, ℨij. three times a-day.

*25th.*—There is little or no alteration. Continue as before.

*26th.*—All the symptoms are considerably relieved, but the discharge, which still remains profuse. Continue.

*28th.*—Complains of nothing now but the discharge, which is rapidly subsiding. Continue.

*Dec. 1st.*—Is quite well. Continue three days longer.

I have now to trust that the success of the above cases, taken from many others of a similar kind, sufficiently show the grounds upon which I was induced to make the present statement.

*Vigo-lane, Burlington-gardens;*
*March 16th,* 1821.

